# Supplementary material for: Reduced representation approaches produce similar results to whole genome sequencing for some common phylogeographic analyses
Source: PLoS One. 2023 Nov 30;18(11):e0291941. doi: 10.1371/journal.pone.0291941 (PMC10688678; doi:10.1371/journal.pone.0291941)
Supplement: S1 Table — (PDF) [file pone.0291941.s001.pdf]

Table S1: AMOVA results. Analysis of the full WGS data did not complete after two weeks on a computing node with 28 cores. WGS values below were analyzed using 100,000 randomly sampled SNPs. *P*-values with an * are significant at α = 0.05.

Marker	Component	DF	Sum Sq	Mean Sq	*P*-value
cytb	Within pops	24	9.63	0.40	0.001*
	Among pops	2	1.92	0.96	0.004*
	Among subspecies	2	1.94	0.97	0.53
	Total	28	13.48	0.48	
mtgenome	Within pops	24	11.23	0.47	0.003*
	Among pops	2	1.21	0.60	0.09
	Among subspecies	2	1.35	0.68	0.32
	Total	28	13.79	0.49	
GBS iPyrad	Within pops	22	47343.47	2151.976	0.001*
	Among pops	2	22299.52	11149.758	0.001*
	Among subspecies	2	38423.40	19211.698	0.19
	Total	26	108066.38	4156.399	
GBS GATK	Within pops	24	63354.97	2639.79	0.001*
	Among pops	2	8580.48	4290.23	0.04*
	Among subspecies	2	12102.63	6051.31	0.15
	Total	28	84038.07	3001.36	
UCE	Within pops	24	2276.03	94.83	0.001*
	Among pops	2	1132.69	566.35	0.001*
	Among subspecies	2	1749.90	874.95	0.25
	Total	28	5158.62	184.24	
WGS	Within pops	24	163663.80	6819.33	0.001*
	Among pops	2	65948.76	32974.38	0.001*
	Among subspecies	2	102953.43	51476.72	0.26
	Total	28	332565.99	11877.36	
