# Supplementary material for: Reduced representation approaches produce similar results to whole genome sequencing for some common phylogeographic analyses
Source: PLoS One. 2023 Nov 30;18(11):e0291941. doi: 10.1371/journal.pone.0291941 (PMC10688678; doi:10.1371/journal.pone.0291941)
Supplement: S2 Table — (PDF) [file pone.0291941.s002.pdf]

Table S2: Pairwise mantel test results among all markers. Values in the matrix represent the correlation coefficient (r^2). Values with an * are significant at $\alpha = 0.001$.

	cytb	mtgenome	GBS iPyrad	GBS GATK	UCE	WGS
cytb		0.9968*	0.5377*	0.5816*	0.9821*	0.4951*
mtgenome			0.5402*	0.5900*	0.9860*	0.4969*
GBS iPyrad				0.8488*	0.6023*	0.9912*
GBS GATK					0.6597*	0.7955*
UCE						0.5645*
WGS						
