# Supplementary material for: Reduced representation approaches produce similar results to whole genome sequencing for some common phylogeographic analyses
Source: PLoS One. 2023 Nov 30;18(11):e0291941. doi: 10.1371/journal.pone.0291941 (PMC10688678; doi:10.1371/journal.pone.0291941)
Supplement: S3 Table — (PDF) [file pone.0291941.s003.pdf]

Table S3: Computational cost associated with each data type. Numbers are from the final analyses and do not include testing different parameters or program settings. Phylogenetics includes maximum likelihood trees for mitochondrial markers and coalescent trees for nuclear markers, each with 100 bootstrap replicates. UCE, cytb, and mtgenome data were developed from whole genome sequencing reads and thus may not reflect cost incurred by the average user. NA: analysis was not applicable to data set; Negligible values are marked with a -.

Marker	Analysis	CPU Time (hours)	Walltime (hours)
cytb	Data Prep	-	-
	ADMIXTURE	NA	NA
	Skyline Plot	9.96	0.36
	**Total**	**9.96**	**0.36**
mtgenome	Data Prep	51.90	1.8
	ADMIXTURE	NA	NA
	Skyline Plot	22.60	0.80
	**Total**	**74.50**	**2.60**
GBS iPyrad	Data Prep	29.78	4.93
	ADMIXTURE	21.11	0.84
	Stairway Plot	44.40	1.59
	**Total**	**95.29**	**7.36**
GBS GATK	Data Prep	41.57	21.07
	ADMIXTURE	18.59	0.95
	Stairway Plot	63.10	2.25
	**Total**	**123.26**	**24.27**
UCE	Data Prep	326.78	30.74
	ADMIXTURE	3.11	0.11
	Stairway Plot	7.96	0.28
	**Total**	**337.85**	**31.13**
WGS	Data Prep	33153.36	1258.41
	ADMIXTURE	11506.00	411.00
	Stairway Plot	197.30	7.05
	**Total**	**44856.66**	**1676.46**
