# Supplementary material for: Reduced representation approaches produce similar results to whole genome sequencing for some common phylogeographic analyses
Source: PLoS One. 2023 Nov 30;18(11):e0291941. doi: 10.1371/journal.pone.0291941 (PMC10688678; doi:10.1371/journal.pone.0291941)

Figure S1: PCA analyses with different iPyrad pipeline settings. A) dataset with filtering as described in main text, B) dataset with subsampling reads for higher coverage individuals, C) dataset using a linkage threshold of 0.3 instead of 0.6, D) dataset using a de novo clustering threshold of 0.85 instead of using a reference genome. SC: South Cascades, NC: North Cascades, NRM: Northern Rocky Mountains, WY: Wyoming, UT: Utah.

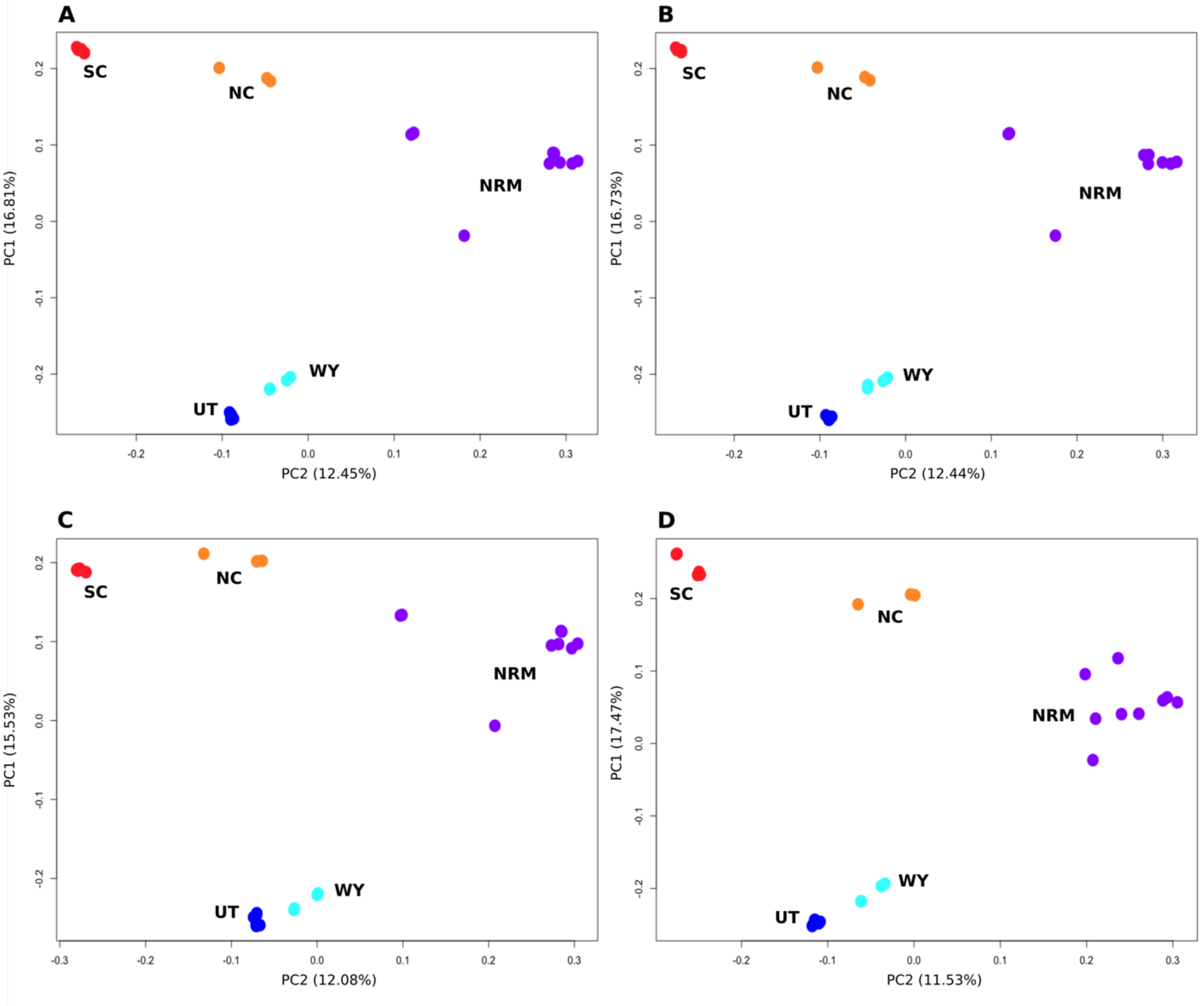

Supplement: S1 Fig — (PDF) [file pone.0291941.s004.pdf]
