# Supplementary material for: Reduced representation approaches produce similar results to whole genome sequencing for some common phylogeographic analyses
Source: PLoS One. 2023 Nov 30;18(11):e0291941. doi: 10.1371/journal.pone.0291941 (PMC10688678; doi:10.1371/journal.pone.0291941)

Figure S2: Venn diagrams displaying the number of shared versus exclusive SNPs among GBS iPyrad, GBS GATK, and WGS datasets.

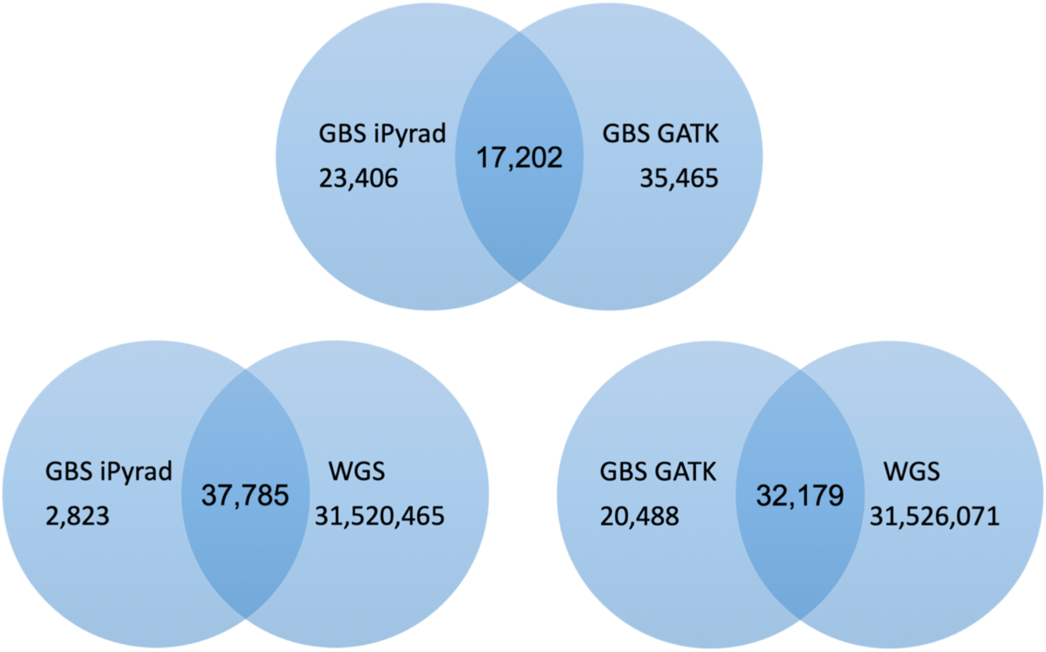

Supplement: S2 Fig — (PDF) [file pone.0291941.s005.pdf]
