# Supplementary figures and images for: Reduced representation approaches produce similar results to whole genome sequencing for some common phylogeographic analyses
Source: PLoS One. 2023 Nov 30;18(11):e0291941. doi: 10.1371/journal.pone.0291941 (PMC10688678; doi:10.1371/journal.pone.0291941)

Figure S3: Full ADMIXTURE results for each nuclear data type for K=3 and K=4.

GBS iPyrad

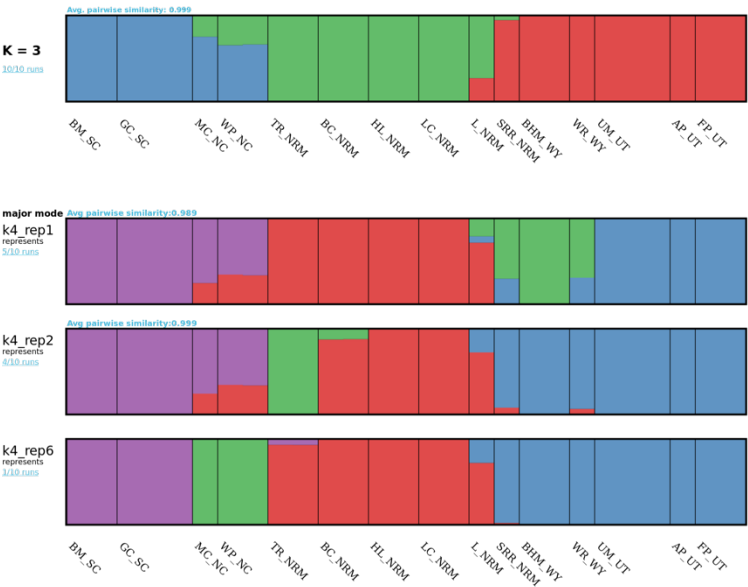

GBS GATK

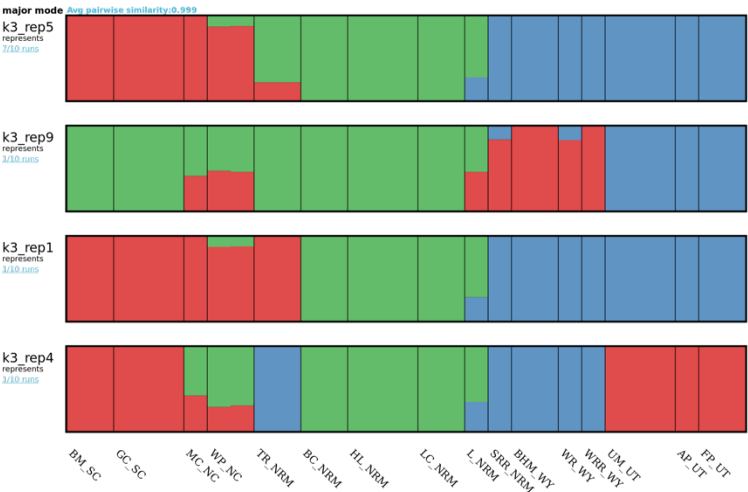

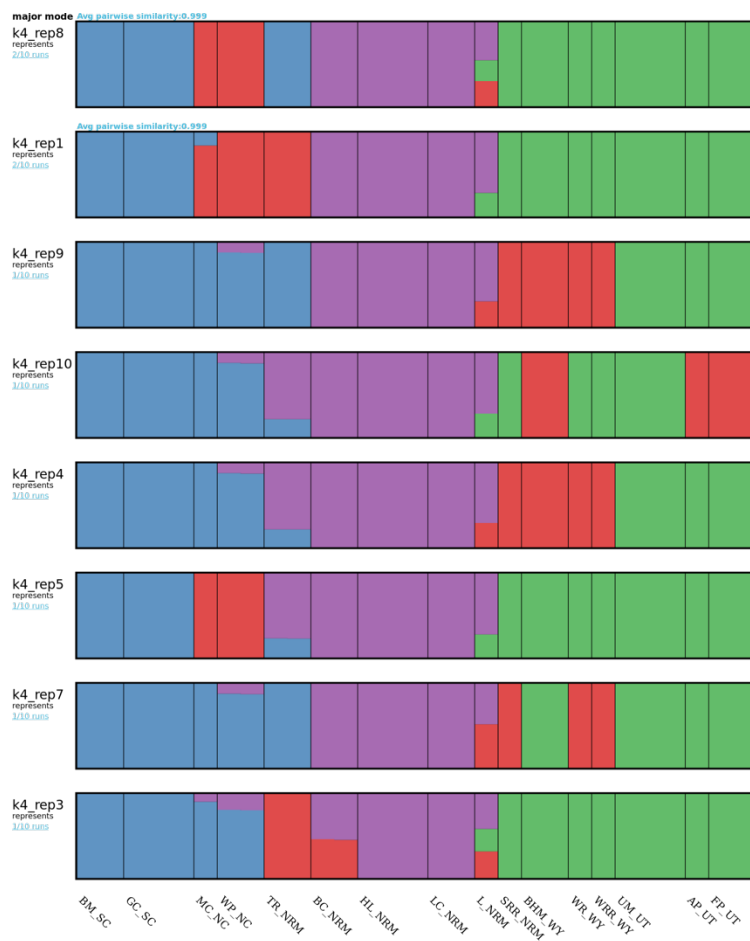

## UCE

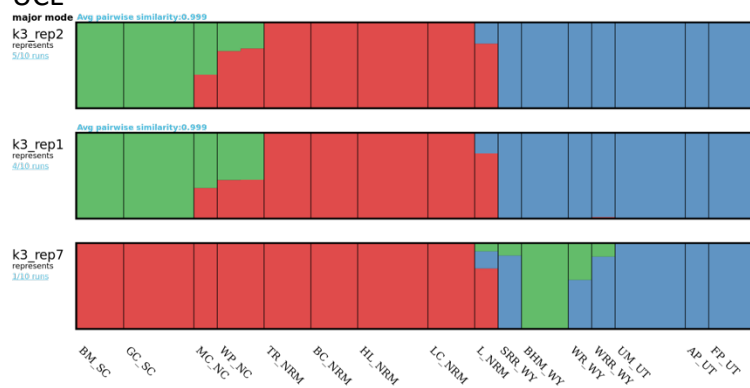

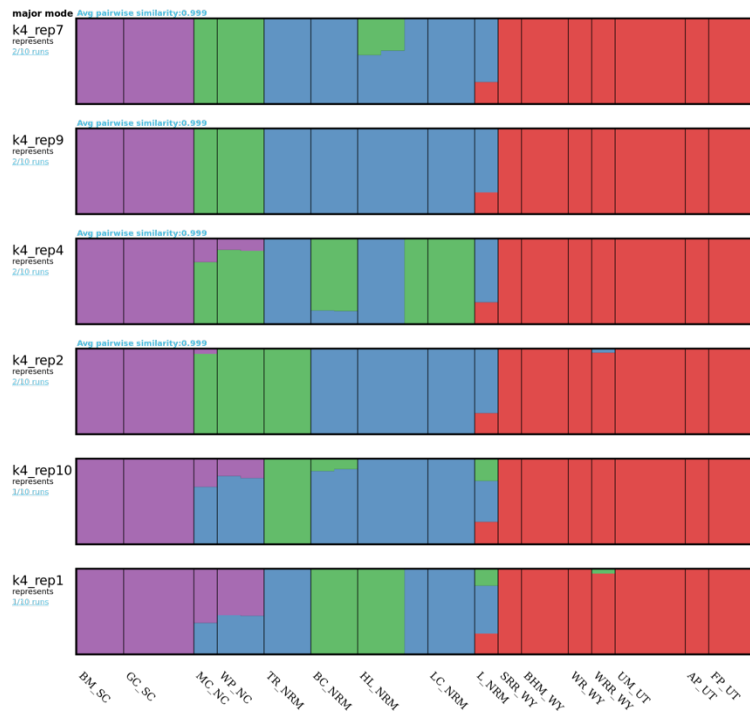

## WGS

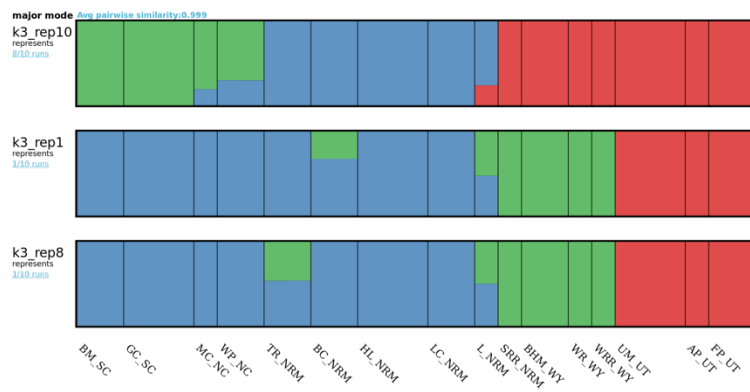

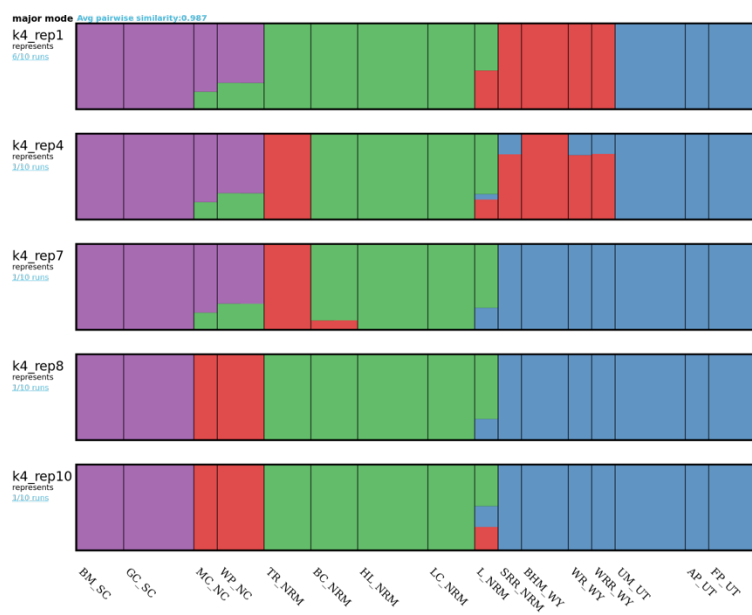

Supplement: S3 Fig — (PDF) [file pone.0291941.s006.pdf]
