# Supplementary material for: Reduced representation approaches produce similar results to whole genome sequencing for some common phylogeographic analyses
Source: PLoS One. 2023 Nov 30;18(11):e0291941. doi: 10.1371/journal.pone.0291941 (PMC10688678; doi:10.1371/journal.pone.0291941)
Supplement: S4 Fig — (PDF) [file pone.0291941.s007.pdf]

Figure S4: Individual Skyline and Stairway plots for each marker type, with confidence intervals.

cytb

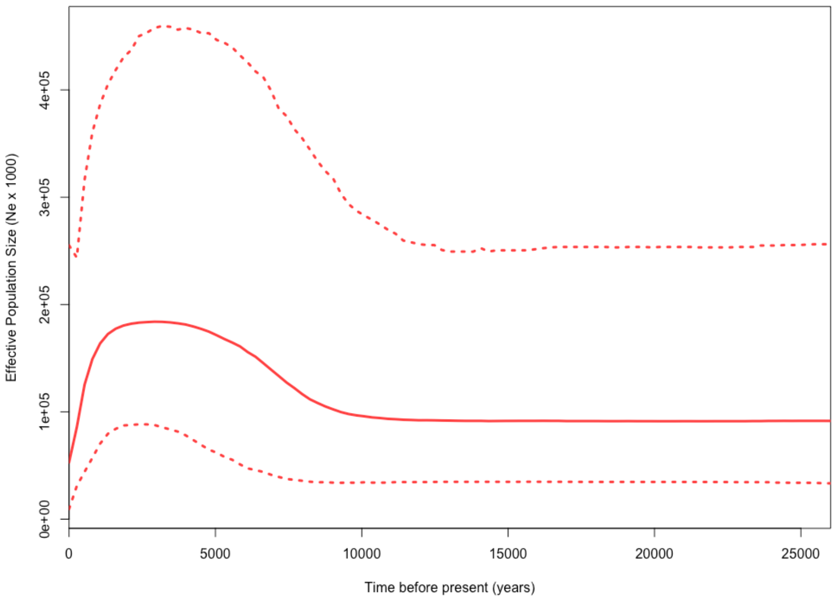

mtgenome

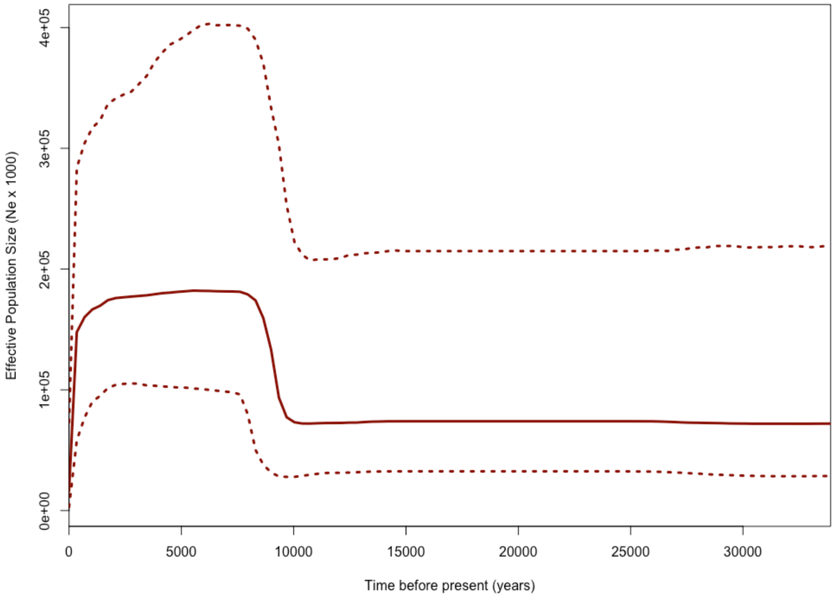

GBS_iPyrad

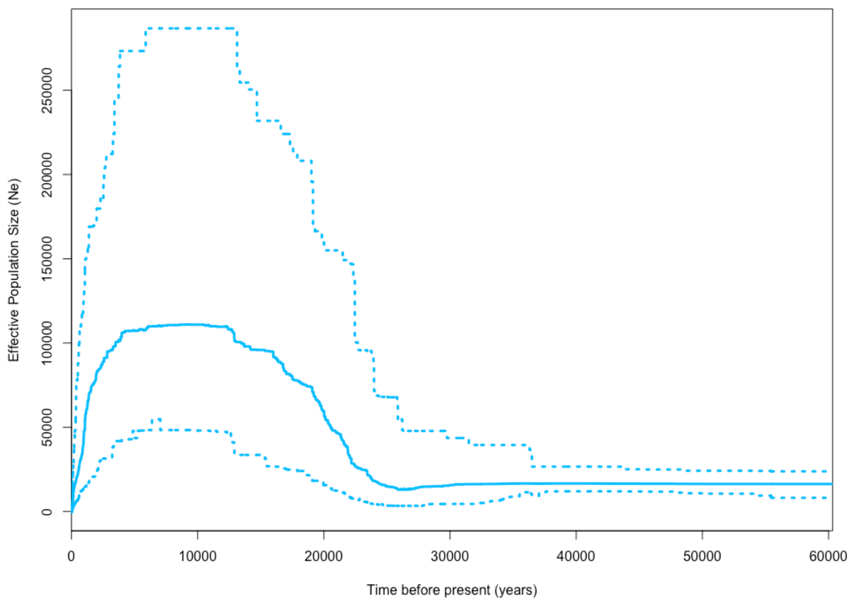

GBS_GATK

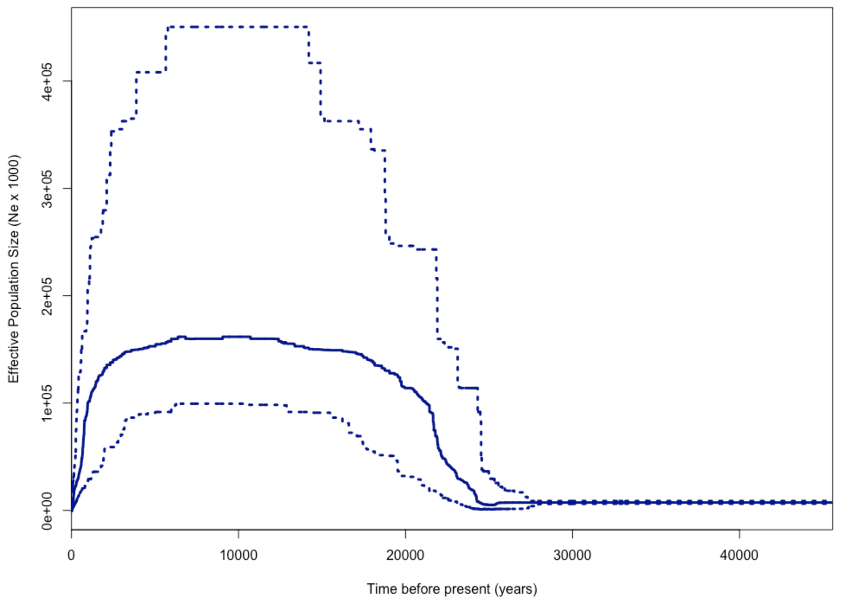

UCE

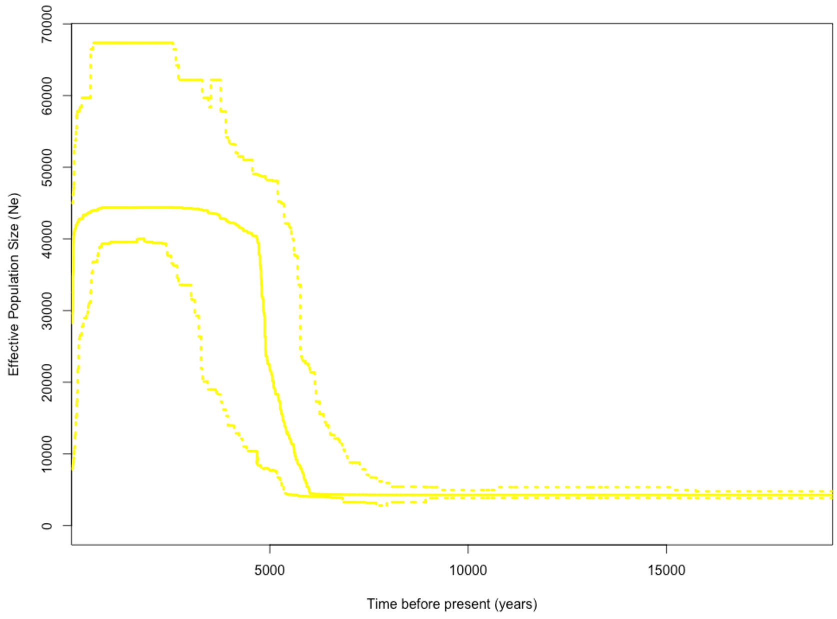
Effective Population Size (Ne)
0
10000
20000
30000
40000
50000
60000
70000
5000
10000
15000
Time before present (years)

WGS

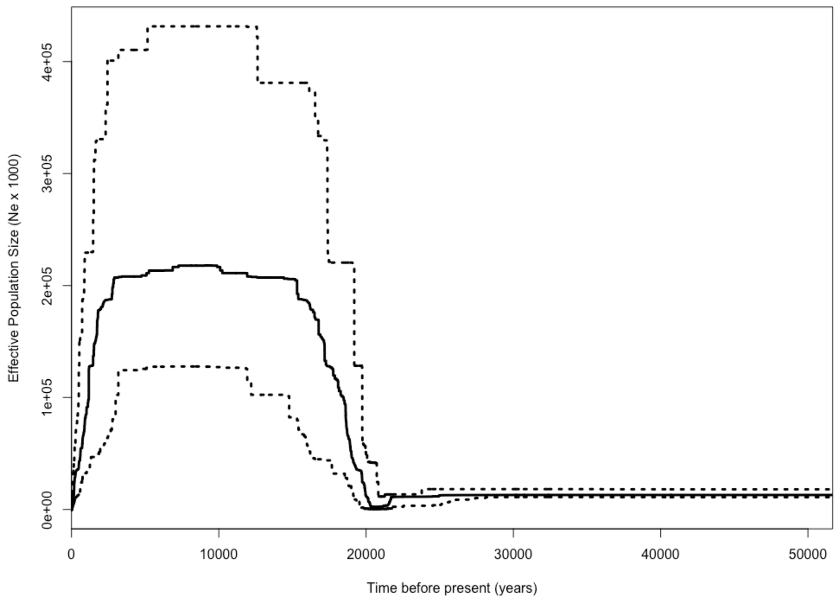
Effective Population Size (Ne x 1000)
0e+00
1e+05
2e+05
3e+05
4e+05
0
10000
20000
30000
40000
50000
Time before present (years)
