# Supplementary material for: Reduced representation approaches produce similar results to whole genome sequencing for some common phylogeographic analyses
Source: PLoS One. 2023 Nov 30;18(11):e0291941. doi: 10.1371/journal.pone.0291941 (PMC10688678; doi:10.1371/journal.pone.0291941)

Figure S5: Maximum likelihood mtgenome phylogeny. SC: South Cascades, NC: North Cascades, NRM: Northern Rocky Mountains, WY: Wyoming, UT: Utah.

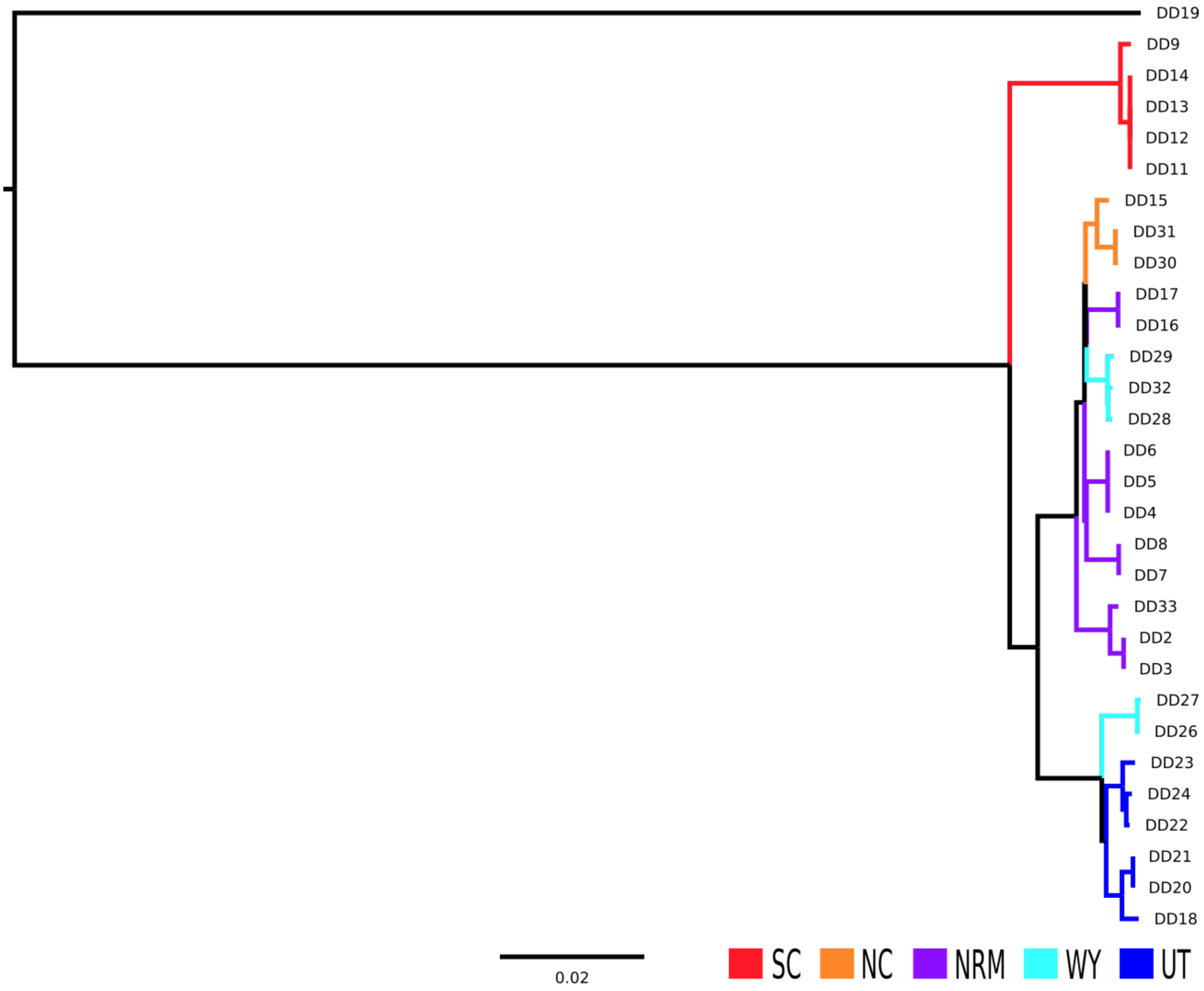

Supplement: S5 Fig — (PDF) [file pone.0291941.s008.pdf]
