# Supplementary material for: Reduced representation approaches produce similar results to whole genome sequencing for some common phylogeographic analyses
Source: PLoS One. 2023 Nov 30;18(11):e0291941. doi: 10.1371/journal.pone.0291941 (PMC10688678; doi:10.1371/journal.pone.0291941)
Supplement: Attachment — Submitted filename: Review_7.7.23.doc [file pone.0291941.s009.doc]

The manuscript entitled “Reduced representation approaches produce similar results to whole genome sequencing for some common phylogeographic analyses” by Duckett et al. compares the results from a set of common phylogeographic 33 analyses using data from mitochondrial Cytochrome b sequences, whole mitochondrial genomes, 34 SNPs isolated via genotyping by sequencing, sequences from ultraconserved elements, and whole 35 nuclear genomes.

The current quality of the manuscript leaves a number of questions to be answered, which are outlined below. The exposition of the results and its discussion in the manuscript needs a major revision, including careful English usage and style edition.

1. In the ‘Abstract’ section, please take care of the English usage. I would suggest that authors use standard English.
2. The ‘Introduction’ section of the manuscript is not coherent. Also, it does not describe the relevance of the study. A few sentences in the ‘Introduction’ are too long and not making any proper sense. Several colloquial words have been used. Please rewrite the ‘Introduction’ section properly in a concise manner.
3. The outcome of the experiments should be described in the ‘Results’ section, and interpretation in consistence with the previous studies should be given in the ‘Discussion’ section. Accordingly, the results and discussion sections should be revised.
4. Within the text, the citations are not arranged properly; few are alphabetically arranged, while others are chronological. Please maintain a uniform pattern.
5. Please revise the ‘Reference’ section thoroughly according to the format.

Overall, the manuscript presentation is not of adequate quality. The English used in the manuscript is not of standard quality. Many colloquial words have been used, which makes the presentation very poor to be published. I would suggest the authors take the help of an English professional. The manuscript should be revised thoroughly for data presentation, result interpretation, description, and language.
