# Supplementary material for: Reduced representation approaches produce similar results to whole genome sequencing for some common phylogeographic analyses
Source: PLoS One. 2023 Nov 30;18(11):e0291941. doi: 10.1371/journal.pone.0291941 (PMC10688678; doi:10.1371/journal.pone.0291941)
Supplement: Attachment — Submitted filename: author response PONE-D-23-05002 reviewer comments.docx [file pone.0291941.s010.docx]

Reviewer #1: This study is a comprehensive look at various types of genomic sequencing and the return on investment for each using a complicated biological system (North American water vole), as an example. I found it thorough, well written and highly informative. I recommend acceptance without revision.

Duckett et al. (DEA). Thank you for your endorsement of our manuscript. We are pleased that you found it to be well written and informative. While we have conducted a number of edits to the writing, particularly in the Introduction and Discussion, we hope that these have resulted in improvements to clarity and ease of understanding for its readers.

Reviewer #2: The current quality of the manuscript leaves a number of questions to be answered, which are outlined below. The exposition of the results and its discussion in the manuscript needs a major revision, including careful English usage and style edition.

DEA. Thank you for your review. We have conducted an extensive edit, particularly of the Introduction and Discussion sections, to improve the quality of the presentation throughout the revised manuscript.

1. In the ‘Abstract’ section, please take care of the English usage. I would suggest that authors use standard English.

DEA. We have rewritten the Abstract to improve clarity. We have attempted to minimize jargon and to use standard English throughout.

2. The ‘Introduction’ section of the manuscript is not coherent. Also, it does not describe the relevance of the study. A few sentences in the ‘Introduction’ are too long and not making any proper sense. Several colloquial words have been used. Please rewrite the ‘Introduction’ section properly in a concise manner.

DEA. We have rewritten the Introduction to improve coherence and to make the relevance of this work more clear. We have replaced many of the words that may have been too colloquial with more technical terms, while endeavoring to maintain readability.

3. The outcome of the experiments should be described in the ‘Results’ section, and interpretation in consistence with the previous studies should be given in the ‘Discussion’ section. Accordingly, the results and discussion sections should be revised.

DEA. We have revised the Results and Discussion sections to describe the outcomes of the experiments in the Results section and the interpretation of these results in light of previous investigations in the Discussion section.

4. Within the text, the citations are not arranged properly; few are alphabetically arranged, while others are chronological. Please maintain a uniform pattern.

DEA. The initial submission included references that were formatted alphabetically. It is our understanding that this is encouraged (or at least allowed) by PLoS ONE (e.g., format free initial submission <https://journals.plos.org/plosone/s/getting-started> ) as a way to minimize the burden on the authors during the initial review process, but we apologize if this is incorrect or if our alphabetically-formated referenced caused undue stress to our reviewers. Regardless, all references are now included in a numerical format following the Vancouver style as described in the author guidelines.

5. Please revise the ‘Reference’ section thoroughly according to the format.

DEA: All references are now included in a numerical format following the Vancouver style as described in the author guidelines.

Overall, the manuscript presentation is not of adequate quality. The English used in the manuscript is not of standard quality. Many colloquial words have been used, which makes the presentation very poor to be published. I would suggest the authors take the help of an English professional. The manuscript should be revised thoroughly for data presentation, result interpretation, description, and language.

DEA: We have taken this opportunity to thoroughly edit this manuscript for clarity and language with the goal of improving the data presentation and interpretation of results. Thank you for your review of our work.
